# Outcomes of Cancer Patients Who Seek Outpatient Visits Despite Post-chemotherapy Decline

**DOI:** 10.7759/cureus.42969

**Published:** 2023-08-04

**Authors:** Hiroaki Goto

**Affiliations:** 1 Oncology and Hematology, Edogawa Hospital, Tokyo, JPN

**Keywords:** outpatient visits, cancer, caregiving capacity, end-of-life care, palliative care, home-based care

## Abstract

Introduction: The medical needs of cancer patients are complex and increase with the disease’s progression. However, Japan’s aging population and increased medical needs have produced challenges like a shortage of hospital beds. Therefore, patients who have completed chemotherapy are recommended home-based care or referred to local palliative care facilities, especially when cancer treatment is no longer a viable option and outpatient visits become unfeasible. However, some patients still strive to continue outpatient visits, which could result in increased pain and possible urgent hospitalization. These patients may spend their final days in the hospital, which may not have been their desired outcome. Therefore, we examined the outcomes of patients who were recommended home-based care.

Methods: The study population comprised 34 cancer patients undergoing treatment at the Oncology Outpatient Department of Edogawa Hospital who were recommended home-based care owing to their difficulty attending outpatient visits. Data regarding home-based care recommendations were obtained from the lists of medical social welfare interventions and hospitalized patients. Patients were classified based on whether they opted for home-based care and their caregivers’ caregiving capacity. Survival was analyzed using the Kaplan-Meier curve and the log-rank test, and the groups were compared using Fisher's exact test.

Results: The median interval between the initial consultation with an oncologist and the date of recommendation for home-based care was six and a half months. Home-based care was mostly recommended because of the progression of cachexia (23 cases) and the worsening of symptoms (11 cases). The median survival for the groups with cachexia and worsening symptoms was 25 days and 35 days, respectively. Ten patients refused home-based care. Of them, six refused it because they wished to continue with outpatient visits. Of the home-based care group (24 patients), only one required an emergency room (ER) visit, while four of the non-home-based care group required ER visits. Regarding end-of-life care, 19 from the home-based care group and four from the non-home-based care group received end-of-life care at home. Additionally, one of the two patients who lived alone and two of the six patients whose caregivers were deemed to have insufficient caregiving capacity received end-of-life care at home. Of the remaining 24 patients, 22 received end-of-life care at home.

Conclusion: The short survival period of the group with cachexia indicates that it would be beneficial for patients, their family members, and home-based care providers to facilitate earlier referral to home-based care. However, while home-based care is beneficial for patients who wish to spend their final days at home and for their family members, short-term and achievable goals, such as attending the next outpatient visit, may alleviate anxiety in cancer patients and enable them to live their daily lives without constant awareness of death. Additionally, while non-home-based care could lead to extended pain due to the lack of readily accessible medical personnel, ER visits, and hospital-based end-of-life care, home-based care can be mentally and physically strenuous for the primary caregiver. Therefore, comprehensive information about palliative care options should be provided early on during the outpatient visits of cancer patients who wish for home-based care for informed decision-making.

## Introduction

The incidence and mortality rates of cancer continue to rise in Japan, primarily due to population aging. However, in terms of age-adjusted rates that exclude the impact of population aging, the cancer incidence rate continuously increased until around 2010 before stabilizing, while the cancer mortality rate has been declining since it peaked in the mid-1990s [1]. Recent advancements in cancer treatment, particularly in early detection and curative resection through treatment, as well as life-prolonging treatment that maintains quality of life (QOL), have resulted in a gradual upward trend in survival rates across many anatomical sites.

Japan's national health insurance system is a social security program that enables all individuals to enroll in health insurance and access necessary healthcare services at an affordable cost. The introduction of this system is credited with contributing to the extension of the average life expectancy in Japan [2]. However, in the face of population aging, challenges, such as rising healthcare costs and a shortage of healthcare professionals, have emerged.

In the case of Japan, the baby boom refers to the phenomenon of a surge in the birth rate during the country's period of rapid economic growth following World War II. Individuals born during this time constitute a large generation that continues to have a significant impact on Japan’s aging society. This has resulted in a shortage of hospital beds. As the population has aged and medical needs have increased, the number of inpatients has grown, leading to a situation in which the limited availability of hospital beds constrains patient admissions. Consequently, waiting times for hospitalization have been extended, and in some cases, patients have had to be transferred to remote locations. Given that the medical needs of older adults are projected to continue to rise, there is a growing demand for the expansion of medical services beyond hospitals, including the development of community-based comprehensive care systems and the promotion of home-based medical care [3].

Cancer patients' medical needs are known to increase as the disease progresses [4]. In addition to the management of complex and advanced treatments and their adverse events, the need for medications, such as analgesics, that are used for symptom relief and supportive care tends to increase. The emergence of symptoms due to physical decline and the spread of the disease must also be considered. The availability of hospital outpatient slots is limited, and these slots tend to be filled by cancer patients receiving chemotherapy. Patients who have completed chemotherapy are recommended home-based care or are referred to local palliative care facilities.

Edogawa Hospital is an established regional general hospital situated in downtown Tokyo. Several generations of patients have used the hospital's services. Many patients have developed the habit of visiting the hospital for any medical concerns. Owing to the availability of affordable healthcare in Japan, many patients desire to receive specialized medical care until their final moments, which has resulted in an overwhelmingly higher percentage of patients receiving hospital-based end-of-life care in comparison to those receiving home-based end-of-life care [5]. However, with the recent increase in the elderly population, it has become increasingly challenging for everyone to receive end-of-life care at a hospital. Similarly, it has become increasingly difficult for patients at Edogawa Hospital who have completed cancer treatment to continue outpatient visits as their medical needs increase. Consequently, an increasing number of patients are advised to obtain home-based care or consult with local palliative care facilities or residential care facilities.

However, when cancer treatment is no longer a viable option and the patients and their family members are likely in a state of shock from the news, the attending physician may find it difficult to inform them that further outpatient visits will be discontinued [6]. Understandably, patients and their family members may find it difficult to accept the fact that they can no longer visit the hospital. Consequently, they may continue visiting crowded outpatient facilities while they are still capable of it.

As the cancer continues to progress, patients may experience a gradual decline in appetite, increased pain, and loss of muscle strength, leading to a state known as irreversible cachexia [7]. Under such circumstances, their condition may change on a weekly basis. There is a risk that their condition may deteriorate before their next scheduled outpatient appointment, necessitating an emergency room (ER) visit or urgent hospitalization. Moreover, it may become difficult for them to attend outpatient appointments, leading to concerns about a shortage of analgesics.

When the decision is made to discontinue cancer treatment, home medical care is presented as an option for patients to spend their final days at home without being hospitalized. Although many patients and their family members find comfort in the existence of such a system, they often do not immediately initiate it. This may be due to the prevalent perception in Japan that hospital visits are the norm and the negative association of home-based care with end-of-life care. As such, in the outpatient department, the progression of the patients' cachexia is monitored, and the difficulty of hospital visits and the need for immediate action are assessed. When it is determined that hospital visits have become increasingly challenging, the option of home-based care is revisited. When patients themselves feel that it has become difficult to continue outpatient visits, many opt for home-based care. However, some patients still strive to continue their hospital visits. Consequently, in some instances, patients endure pain at home and eventually require an ER visit or urgent hospitalization. These patients may ultimately spend their final days in the hospital, which raises questions about how home-based care could have been recommended more effectively. In this study, we examined the outcomes of patients who were advised to consider home-based care after it was determined that continuing hospital visits would be challenging.

## Materials and methods

The study population comprised cancer patients undergoing treatment at the Oncology Outpatient Department of Edogawa Hospital. In our analysis, we included patients for whom physicians determined that further outpatient management would be challenging and therefore recommended home-based care. The study period spanned from January 1 to December 31, 2022. The data were retrospectively analyzed using an electronic medical record system.

When home-based care is proposed, medical social welfare counselors (MSWs) are always involved in explaining the home-based healthcare system, associated costs, and the process of enrollment into the long-term care insurance system. Therefore, we referred to the list of cases in which MSWs intervened. When a patient refuses the recommendation of home-based care, there is no intervention by MSWs. In such cases, patients whose condition has changed are hospitalized. We checked the list of hospitalized patients to confirm whether home-based care had been recommended during their outpatient visits.

Home-based care may be proposed as an option at the conclusion of treatment or when treatment is nearing completion. However, such cases were not included in the analysis. We only included cases in which the physician determined that it would be difficult for the patient to attend the next outpatient visit and subsequently recommended home-based care.

The assessment of caregiving capacity is based on the physician’s experience in determining whether the caregiver is capable of fulfilling his/her duties until end-of-life care. Although there is no formal definition, factors such as the caregiver’s age, pre-existing conditions, and ability to perform activities of daily living are taken into consideration. For instance, if the caregiver is an older adult with dementia who is also receiving care, it can be inferred that providing care would be challenging for the caregiver. This is because terminal cancer patients have difficulty accurately communicating their symptoms to visiting nurses and physicians. Therefore, caregivers need to do this on their behalf. Moreover, caregivers need to possess a deep understanding of nocturnal delirium symptoms, and it can be challenging to deal with the agitation or difficult behavior exhibited by patients. We, the authors of this paper, determined the presence or absence of caregiving capacity based on our experience in home-based care, given that we make weekly home visits.

In this study, survival was defined as the duration from the date of recommendation for home-based care in an outpatient setting until the date of death. The date of death following the transition to home-based care was determined by referring to reports received at a later date from the facility that provided home-based care. Survival was analyzed using the Kaplan-Meier curve and the log-rank test. Comparisons between two unrelated groups were conducted using Fisher's exact test. All statistical analyses were performed using EZR, a statistical software tool that extends the functionality of R and R-Commander.

## Results

There were 34 eligible patients with a median age of 74 years (range: 30-86); 16 were male and 18 were female. All patients were stage IV. By cancer type, there were nine patients with breast cancer, eight with pancreatic cancer, four with gastric cancer, three with lung cancer, three with cancer of unknown primary, two with colorectal cancer, and five patients with one of the following types of cancer: prostate cancer, bladder cancer, bile duct cancer, head and neck cancer, and peritoneal mesothelioma (Table 1).

**Table 1:** Patients’ characteristics

Characteristic	n=34
Age, years, median(range)	74 (30–86)
Sex, n (%)	
Male	16 (47.1)
Female	18 (52.9)
Cancer type, n (%)	
Breast	9 (26.5)
Pancreatic	8 (23.5)
Gastric	4 (11.8)
Lung	3 (8.8)
Primary unknown	3 (8.8)
Colorectal	2 (5.9)
Others	5 (14.7)

The median interval between the date of cancer diagnosis and the date of recommendation for home-based care was 21 months (ranging from one to 132 months). In particular, the median interval between the initial consultation with an oncologist and the date of recommendation for home-based care was six and a half months (ranging from zero to 47 months). The median number of administered chemotherapy regimens was two (ranging from zero to seven). Home-based care was recommended in 31 cases (91.2%) in an outpatient setting, while in three cases (8.8%), it was recommended during a telephone consultation while the patients were at home awaiting their outpatient visits.

**Figure 1:**
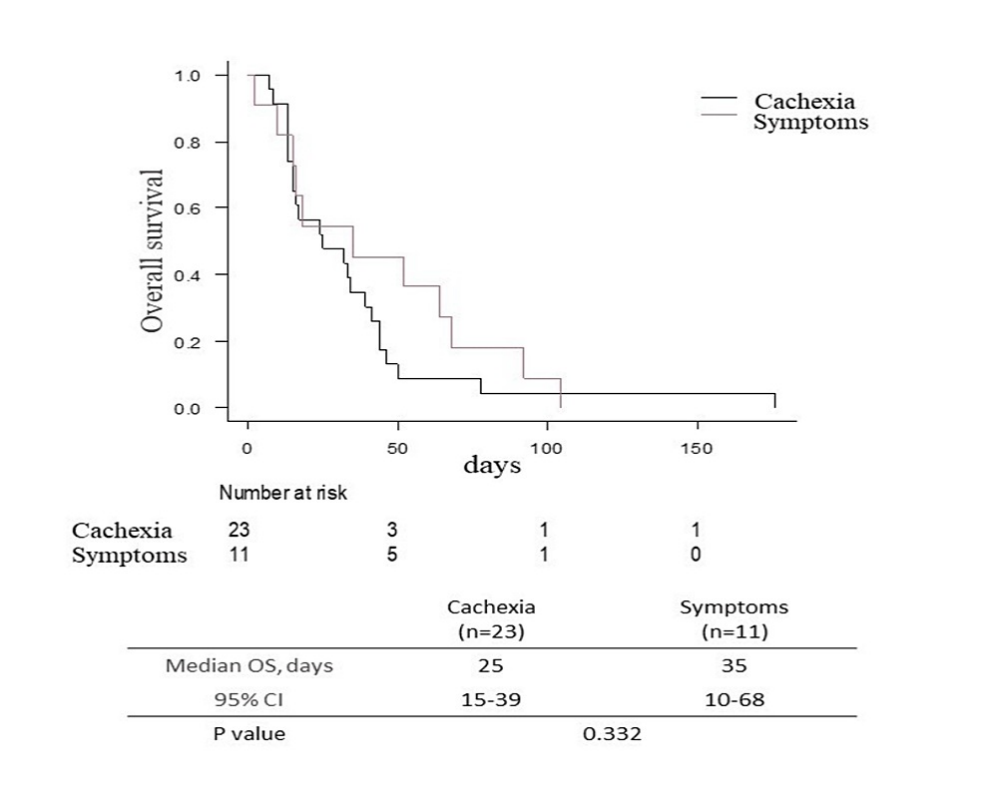
Median survival for the group with cachexia and symptoms

The most common reason for physicians recommending home medical care was the progression of cachexia (23 cases; 67.6%), followed by worsening of symptoms (11 cases; 32.4%). The judgment of cachexia was not based on criteria but on what was written in the medical record from clinical experience. The survival curves for these two groups (Figure 1) indicated that the median survival for the group with cachexia was 25 days (95% confidence interval [CI]: 15-39 days), while for the group with worsening symptoms, it was 35 days (95% CI: 10-68 days). Although the log-rank test on the Kaplan-Meier survival curves showed no significant difference (p=0.332), the survival curve for the group with cachexia exhibited a rapid decline, indicating a poor prognosis with a life expectancy of less than one month.

Of the patients who were recommended home-based care during an outpatient visit, 24 (70.6%) followed the suggestion, while 10 (29.4%) refused. The most common reason for refusal was the desire to persevere with outpatient visits, accounting for six cases (60%), followed by opposition to having medical personnel enter their home (two cases; 20%) and other reasons (two cases; 20%). As shown in Table 2, a comparison of the proportion of patients with emergency room (ER) visits between the home-based care group and the non-home-based care group revealed that only one of the 24 patients (4.1%) in the home-based care group required an ER visit, compared with four of the 10 patients (40%) in the non-home-based care group, with a significant difference at p = 0.019. Of the five patients who had an ER visit, four were hospitalized as emergencies. Of these, two received end-of-life care in the hospital, while the other two were discharged to initiate home-based care and subsequently received end-of-life care at home.

**Table 2:** A comparison of the proportion of patients with emergency room visits

	Non-ER visit	ER visit	
Home-based care	23	1	
Non-home-based care	6	4	p=0.019

Table 3 compares the proportion of patients who received end-of-life care at home between the home-based care group and the non-home-based care group. Nineteen of the 24 patients (79.2%) in the home-based care group received end-of-life care at home, compared with six of the 10 patients (60%) in the non-home-based care group. Although there was no significant difference at p = 0.395, there was a trend toward a higher proportion of end-of-life care received at home among those who initiated home-based care as soon as it was recommended.

**Table 3:** A comparison of end-of-life care locations and home-based care interventions

	End-of-life care		P-value
	At home	At hospital	
Home-based care	19	5	
Non-home-based care	6	4	0.395

The presence of a caregiver is important for receiving and continuing home-based care and for completing end-of-life care at home. Out of all 34 patients, two (5.9%) lived alone without a caregiver; one of them received end-of-life care at home, while the other received end-of-life care at the hospital. An analysis of the caregivers for the remaining 32 patients is shown in Table 4. The proportion of patients who received end-of-life care at home was 33.3% (two out of six patients) among those whose caregivers were deemed by the physician to have insufficient caregiving capacity, while it was 84.6% (22 out of 26 patients) among those whose caregivers were deemed to have sufficient caregiving capacity, with a significant difference at p=0.0228.

**Table 4:** A comparison of caregiver capacity and end-of-life care locations

Caregiving capacity	End-of-life care		p-value
	At home	At hospital	
Insufficient	2	4	
Sufficient	22	4	0.0228

## Discussion

Our findings showed that the median interval from the initial consultation with an oncologist to the recommendation of home-based care was a brief six and a half months. Although patients with various types of cancer were included in the analysis, considering recent advances in chemotherapy, it is noteworthy that patients became unable to continue outpatient visits within approximately six months of initiating treatment. Breast and pancreatic cancers accounted for half of the total cases. Chemotherapy for breast cancer in Japan is often performed by breast surgeons, even after recurrence. A consultation with a medical oncologist is often conducted when symptoms occur or when other interventions besides chemotherapy become necessary. Therefore, it is inferred that the intervals from medical oncologist consultation to treatment resistance are relatively short. Pancreatic cancer also has a poor prognosis, but recent advances in chemotherapy have led to prolonged survival [8]. In Japan, the incidence of pancreatic cancer has been increasing, even after adjusting for age [1]. Elderly patients and those diagnosed at an advanced stage may not benefit from new treatments and may experience rapid deterioration of their condition. Pancreatic cancer is also known for its tendency to progress to cachexia [9], which likely made outpatient visits difficult at an early stage.

This study included only a few cases of lung and colorectal cancers, which have high incidence rates in Japan. The relatively slow progression of these cancers and remarkable advances in treatment may have reduced the likelihood of rapid deterioration of patients' conditions [10,11], and patients are less likely to be recommended urgent initiation of home-based care if their condition does not deteriorate rapidly. If a patient contracts an infectious disease (e.g., pneumonia or bedsores) or experiences a pathological change in condition, such as jaundice or bleeding, during outpatient care, the patient is likely to require an ER visit or urgent hospitalization before the next scheduled appointment. Subsequently, a transition to home-based care may be suggested during the patient's hospital stay.

For many patients, cachexia was cited as the reason it became difficult to make outpatient visits. Although the number of cases was insufficient to reach statistical significance, the survival curve for the cachexia group declined rapidly, with a survival period of less than one month. In Japan, home-visit medical and nursing services for terminal cancer patients typically include four visits per week by healthcare professionals, with possible additional visits in case of an emergency. Typically, a physician's visit comprises one of the four weekly visits. This means that patients who initiated home-based care because of cachexia received only approximately four visits, including the initial visit. Given the necessity of multiple face-to-face interactions with patients and their family members to establish a relationship of trust, it can be surmised that many patients pass away before such a relationship can be established. Furthermore, the time available for patients to fulfill their wishes and arrange their affairs while receiving palliative care at home may be insufficient under these circumstances. These findings indicate that it would be beneficial for patients, their family members, and home-based care providers to facilitate earlier referral to home-based care.

While many patients accepted their physician's recommendation for home-based care, some did not. A large-scale survey of bereaved families in Japan on the concept of a "good death" revealed the importance of factors such as trust in their physician and the ability to live without constant awareness of their illness or impending death [12]. It is natural for patients who have established a relationship of trust with their treating physician to want to consult with them when faced with difficulties. Additionally, having short-term and achievable goals, such as returning for the next outpatient visit, may alleviate anxiety in cancer patients and enable them to live their daily lives without constantly being conscious of death. Recommending home-based care is undoubtedly a valuable suggestion for patients who wish to spend their final days at home and for their family members. Considering that some patients wish to persevere with outpatient visits and that their family members wish to support them in this endeavor, healthcare professionals should understand that disregarding the patients' option to not immediately initiate home-based care may lead to a loss of hope for the patients.

Compared with patients who immediately initiated home-based care, a significantly higher proportion of patients who declined home-based care had an ER visit. An ER visit indicates that the patient experienced severe distress at home. Home-based care provides 24-hour access to medical personnel, often a visiting nurse, which enables patients to get relief from distress in their own homes. In contrast, without home-based care, patients experiencing severe distress at home must contact the hospital and may require an ambulance. Upon arrival at the hospital, various tests must be performed, resulting in further delays. Even after the pain assessment is completed, the on-call physician may not be well-versed in cancer pain treatment, which can prolong the patient's suffering. If a patient visits the ER in such a state, it may be natural for the doctor to decide against immediate discharge. Moreover, family members who have seen the patient suffer at home may feel apprehensive about the patient returning home again. In other words, while a patient's desire to persevere with hospital visits should not be denied, it may result in the patient enduring intense pain for extended periods and leading to an emergency admission to a hospital, an outcome that the patient would not otherwise choose. Furthermore, it could lead to the patient spending their final days in hospital settings owing to their family members' apprehension about the patient returning home. Patients and their family members should be aware of these potential outcomes when choosing a place of treatment.

A comparison of the patients who received end-of-life care at home showed that the proportion of these patients tended to be higher in the group that initiated home-based care, although no significant difference was observed. However, not all patients who initiated home-based care were necessarily cared for at home. We examined how the presence or absence of a caregiver and their caregiving capacity influenced whether a patient received end-of-life care at home. Although only two of the 34 patients lived alone, it may be important to note that neither was eventually hospitalized. If a robust support system that considers patients' wishes and utilizes social resources is established, even those who live alone may be able to spend their final days in the comfort of their own homes. Furthermore, even in cases in which a caregiver is present, providing physically demanding care may prove difficult if the caregiver is an older adult or has a pre-existing condition related to the musculoskeletal or cardiovascular systems [13]. Despite the 24-hour availability of emergency support from professional caregivers and visiting nurses, the primary caregiver is often responsible for attending to the patient throughout most of the day. For instance, at home, a wife may need to accompany her husband to the toilet at all times until the end if he strongly desires to defecate in the toilet. Alternatively, if the patient defecates in a diaper while in bed, regular repositioning and diaper changes are necessary. If the patient experiences delirium during the terminal stage, the caregiver may be constantly occupied with addressing the patient's complaints of pain and unintelligible demands, regardless of the time of day. Therefore, there is a concern that the caregiver may become mentally and physically exhausted owing to prolonged sleep deprivation. We established that when physicians determined that caregivers lacked adequate caregiving capacity, the proportion of patients who received end-of-life care at home was significantly lower compared with when caregivers were deemed to possess adequate caregiving capacity. Considering this, when a physician determines that a caregiver lacks adequate caregiving capacity, it is important for healthcare professionals, the patient, and family members to discuss the patient's desire and need for hospitalization while continuing home-based care. This can help prevent caregivers from becoming overwhelmed and allow for early consultation with a hospital that provides end-of-life care, such as a hospice or palliative care ward. In some cases, it may also be necessary for the hospital to provide the patient with information about hospices and palliative care wards while the patient is still making outpatient visits and to contact a facility that provides such end-of-life care on behalf of the patient while simultaneously referring the patient to home-based care.

Study limitations

As I, the author, am both a medical oncologist and a palliative care specialist, it is possible for me to continue providing palliative treatment on an outpatient basis even after a patient completes chemotherapy. Therefore, the patients could continue to receive outpatient care up until the point when it became too difficult for them to continue outpatient visits. In contrast, oncologists without specialized training in palliative care may have advised home-based care at an earlier stage than the authors, upon completion of treatment or worsening of symptoms. Furthermore, because of a lack of knowledge about home-based care among treating physicians, they may not provide detailed information about the option of home-based end-of-life care and instead recommend hospital-based end-of-life care when the patient's condition worsens and their medical needs increase, as the physician could believe that home-based medical care may not adequately address those needs. Therefore, they may recommend hospital-based end-of-life care without thoroughly presenting the option of home-based end-of-life care.

During the outbreak of the novel coronavirus in Japan, many hospitals prohibited visits by family members of hospitalized patients [14]. Temporary outings and overnight stays outside the hospital were also prohibited owing to the associated risk of infection. Therefore, it is plausible that the inability of patients and their family members to spend the final moments together resulted in an increased acceptance of home-based care during the pandemic. However, with the recent revision (May 2023) of Japan’s Infectious Diseases Control Law eliminating the previous mandatory isolation requirements, if restrictions on hospital visits by family members and overnight stays outside the hospital are lifted, there may be a decline in the number of patients initiating home-based care. Therefore, more detailed information about home-based care needs to be provided early on during the outpatient visits of cancer patients who wish to receive care at home.

## Conclusions

In this study, we analyzed cases in which home care was recommended to cancer patients who continued outpatient visits after completion of their cancer treatment but experienced difficulty with further outpatient visits owing to a decline in their condition or worsening of symptoms. Many of these patients eventually accepted home-based care, which preceded end-of-life care at home. However, we found that some patients who still wish to continue outpatient visits may experience a deterioration in their condition and eventually require an ER visit. Many of these patients may then require urgent hospitalization, and they may be unable to return home again. While understanding a patient's desire to persevere with outpatient visits, we also discussed the possibility that the patient may not be able to return home as a result of overexertion. Going forward, we aim to conduct further research to determine the most desirable form of end-of-life care.

## References

[1] (2023). Cancer information. https://ganjoho.jp/public/index.html.

[2] (2023). Japanese medical insurance system. https://www.med.or.jp/people/info/kaifo/.

[3] (2023). The approach to healthcare delivery in an aging society centered around the baby boomer generation. https://www.jmari.med.or.jp/download/WP322.pdf.

[4] Hart NH, Crawford-Williams F, Crichton M (2022). Unmet supportive care needs of people with advanced cancer and their caregivers: A systematic scoping review. Crit Rev Oncol Hematol.

[5] (2023). Recent trends in home medical care. https://www.mhlw.go.jp/seisakunitsuite/bunya/kenkou_iryou/iryou/zaitaku/dl/h24_0711_01.pdf.

[6] Berkey FJ, Wiedemer JP, Vithalani ND (2018). Delivering bad or life-altering news. Am Fam Physician.

[7] Fearon K, Strasser F, Anker SD (2011). Definition and classification of cancer cachexia: an international consensus. Lancet Oncol.

[8] Kolbeinsson HM, Chandana S, Wright GP, Chung M (2023). Pancreatic cancer: a review of current treatment and novel therapies. J Invest Surg.

[9] Baracos VE, Martin L, Korc M, Guttridge DC, Fearon KC (2018). Cancer-associated cachexia. Nat Rev Dis Primers.

[10] Piawah S, Venook AP (2019). Targeted therapy for colorectal cancer metastases: a review of current methods of molecularly targeted therapy and the use of tumor biomarkers in the treatment of metastatic colorectal cancer. Cancer.

[11] Wang M, Herbst RS, Boshoff C (2021). Toward personalized treatment approaches for non-small-cell lung cancer. Nat Med.

[12] Hirai K, Miyashita M, Morita T, Sanjo M, Uchitomi Y (2006). Good death in Japanese cancer care: a qualitative study. J Pain Symptom Manage.

[13] Thana K, Lehto R, Sikorskii A, Wyatt G (2021). Informal caregiver burden for solid tumour cancer patients: a review and future directions. Psychol Health.

[14] (2023). Questionnaire survey results on the impact of COVID-19 in palliative care wards. https://www.hpcj.org/info/covid19/covid19_pcuchosa202103.pdf.

